# The effect of one year lifestyle intervention on eGFR in children and adolescents with overweight, obesity and morbid obesity

**DOI:** 10.1038/s41598-019-40767-4

**Published:** 2019-03-14

**Authors:** Mark van Dam, Jesse Rijks, Elke Dorenbos, Flore Horuz, Karin van Dael, Anita Vreugdenhil

**Affiliations:** 1 Centre for Overweight Adolescent and Children’s Healthcare (COACH), Maastricht University Medical Centre, Maastricht, The Netherlands; 2 Department of Paediatrics, Maastricht University Medical Centre, Maastricht, The Netherlands; 3 School of Nutrition and Translational Research in Metabolism (NUTRIM), Maastricht University, Maastricht, The Netherlands

## Abstract

Obesity causes modifications in the kidneys reversed by weight loss in adults. There is little data on renal function and effects of weight loss in children with obesity. The aim of this prospective study was to examine renal function and effect of a lifestyle intervention in children with overweight, obesity and morbid obesity. Two hundred forty-five children (age 12.4 ± 3.3 years, 40% boys, BMI z-score 3.46 ± 0.70) participating in an out-patient lifestyle intervention were included. Children with at least 12 months follow-up (n = 144 (58.8%)) were included in the longitudinal study. Anthropometry, blood analysis and blood pressure measurements were performed at baseline and follow-up. Glomerular filtration rate (GFR) was estimated using the Schwartz and FAS equation. eGFR was de-indexed using body surface area. Different cut-off points for defining glomerular hyperfiltration were used for stratification. Depending on the definition and equation used, glomerular hyperfiltration was present in 2% to 18% of the participants. After intervention, de-indexed eGFR decreased significantly in children with baseline glomerular hyperfiltration, depending on the eGFR equation and definition for glomerular hyperfiltration used. No associations of changes in eGFR with changes in BMI z-score, blood pressure or parameters of glucose and lipid metabolism were found. In conclusion, after one year of lifestyle intervention, eGFR decreases in hyperfiltrating children and adolescents with overweight, obesity and morbid obesity. eGFR and changes over time in children with obesity depend on eGFR equation used and on de-indexing for body surface area.

## Introduction

Obesity is known to be an independent risk factor for the development of chronic kidney disease^1,2^. As a consequence of the increasing prevalence of childhood obesity, the prevalence of obesity-related glomerulopathy (ORG) is likely to increase. Obesity causes structural, haemodynamic and metabolic modifications in the kidneys, that are probably the result of (failed) compensatory mechanisms, leading to ORG^3,4^. ORG is histologically defined by glomerular hypertrophy and focal segmental glomerulosclerosis, that can already be present in children and adolescents with obesity^2,5^. A hallmark of the early phase of ORG is an increase in whole-kidney glomerular filtration rate (GFR). Albeit the pathogenesis of this glomerular hyperfiltration is not completely understood, it is known that glomerular hyperfiltration eventually results in podocyte detachment, proteinuria and progression to chronic kidney disease^2^. Moreover, glomerular hyperfiltration has been found to be an independent risk factor for all-cause mortality in an apparently healthy population^6^.

In adults with obesity and chronic kidney disease weight loss improves proteinuria, albuminuria and normalizes the GFR^7^. Despite extensive research on the effects of weight loss on renal function in adults with obesity, to the best of our knowledge, no research has investigated these effects in children. Therefore, we conducted a prospective study to assess the effect of one year of interdisciplinary lifestyle intervention on renal function in children with overweight, obesity and morbid obesity and the intermediary role of anthropometric changes and cardiovascular risk markers.

## Results

**Figure 1:**
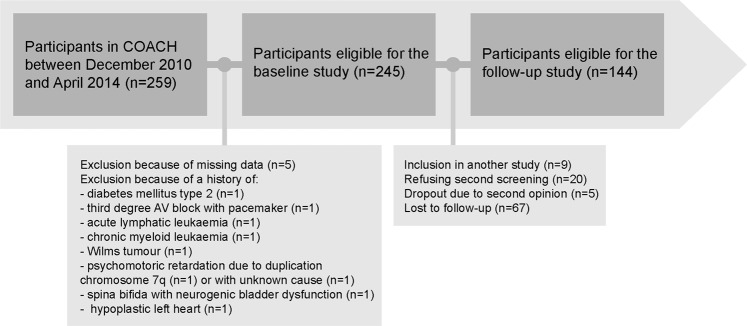
Flowchart of study inclusion. Abbreviations: COACH, Centre for Overweight Adolescent and Children’s Healthcare.

245 children with a mean age of 12.4 ± 3.3 years were included for analyses in the baseline study, as presented in Fig. 1. Baseline characteristics are presented in Table 1. 40% of the subjects were boys. Mean baseline BMI z-score was 3.46 ± 0.70. 14.3% of children were classified as overweight, 42.9% as obese, and 42.9% as morbidly obese. Glomerular hyperfiltration was present in 2% to 18% of the participants, depending of the definition and equation used. Seven participants (2.9%) presented with microalbuminuria.

**Table 1:** Baseline characteristics of the total cohort and stratified according to non-dropout versus dropout after one year lifestyle intervention.

Variable	Total (n = 245)	Non-dropout (n = 144)	Dropout (n = 101)	*P*-value
Age (years)	12.4 ± 3.3	12.0 ± 3.0	13.0 ± 3.5	0.025*
Boys (%)	40	45	34	0.09
BMI z-score	3.46 ± 0.70	3.45 ± 0.65	3.48 ± 0.78	0.76
BSA (m²)	1.86 ± 0.46	1.81 ± 0.42	1.93 ± 0.50	0.06
Overweight (%)	14.3	11.1	18.8	0.10
Obese (%)	42.9	48.6	34.7	0.04*
Morbid obese (%)	42.9	40.3	46.5	0.36
Prepubertal (%)	30.3	28.9	31.8	0.72
Peripubertal (%)	20.4	26.3	13.6	0.09
Postpubertal (%)	49.3	44.7	54.5	0.31
Waist circumference z-score	5.7 (1.4–13.9)	5.5 (1.4–13.9)	5.7 (1.8–11.5)	0.46
Hip circumference z-score	4.3 ± 2.0	4.3 ± 1.9	4.4 ± 2.2	0.60
Waist-to-hip ratio	0.93 ± 0.09	0.93 ± 0.07	0.93 ± 0.10	0.99
Systolic BP z-score	0.3 ± 1.1	0.3 ± 1.0	0.3 ± 1.2	0.91
Diastolic BP z-score	−0.4 ± 1.2	−0.4 ± 1.0	−0.4 ± 1.4	0.92
eGFR-Schwartz (ml/min/1.73 m²)	117.4 ± 20.5	119.6 ± 19.9	114.3 ± 21.1	0.06
eGFR-Schwartz > 135 ml/min/1.73 m² (%)	18.0	22.2	12.0	0.04*
eGFR-Schwartz > 140 ml/min/1.73 m² (%)	12.7	15.3	8.9	0.17
eGFR-Schwartz > 150 ml/min/1.73 m² (%)	5.7	6.9	4.0	0.41
eGFR-Schwartz de-indexed (ml/min)	124.2 ± 30.8	123.5 ± 28.0	125.2 ± 34.4	0.66
Serum creatinine (mg/dL)/Q value	0.97 ± 0.15	0.96 ± 0.14	0.98 ± 0.16	0.32
eGFR-FAS (ml/min/1.73 m²)	113.3 ± 17.1	113.9 ± 16.3	112.3 ± 18.3	0.47
eGFR-FAS > 135 ml/min/1.73 m² (%)	11.4	13.0	9.9	0.68
eGFR-FAS > 140 ml/min/1.73 m² (%)	6.9	6.9	6.9	0.99
eGFR-FAS > 150 ml/min/1.73 m² (%)	2.0	1.4	3.0	0.41
eGFR-FAS de-indexed (ml/min)	121.4 ± 34.6	118.9 ± 30.5	124.9 ± 39.5	0.18
UACR (mg albumin/g creatinine)	4.4 (0–203.3)	4.0 (0.0–36.2)	4.4 (0.0–203.3)	0.10
Microalbuminuria (%)	2.9	2.1	4.0	0.45
Fasting glucose (mmol/L)	4.1 (2.1–5.7)	4.0 (2.5–5.6)	4.1 (2.1–5.7)	0.003*
Fasting insulin (pmol/L)	15.3 (2.0–111.2)	15.0 (2.0–111.2)	15.5 (3.0–76.8)	0.40
HOMA-IR	2.5 (0.4–19.3)	2.5 (0.4–19.3)	2.8 (0.5–18.4)	0.16
HbA1c (%)	5.3 ± 0.4	5.3 ± 0.4	5.3 ± 0.5	0.25
Total cholesterol (mmol/L)	4.5 ± 0.8	4.6 ± 0.8	4.3 ± 0.9	0.03*
HDL-cholesterol (mmol/L)	1.2 ± 0.3	1.2 ± 0.3	1.2 ± 0.3	0.67
LDL-cholesterol (mmol/L)	2.7 ± 0.7	2.8 ± 0.7	2.6 ± 0.7	0.08
Triacylglycerides (mmol/L)	1.0 (0.4–3.7)	1.1 (0.4–3.7)	1.0 (0.4–3.1)	0.20

Abbreviations: BMI, body mass index; BSA, body surface area; BP, blood pressure; eGFR, estimated glomerular filtration rate; FAS, full age spectrum; UACR, urine albumin-to-creatinine ratio; HOMA-IR, homeostatic model assessment of insulin resistance; HbA1c, glycated hemoglobin; HDL, high density lipoprotein; LDL, low density lipoprotein.

**Figure 2:**
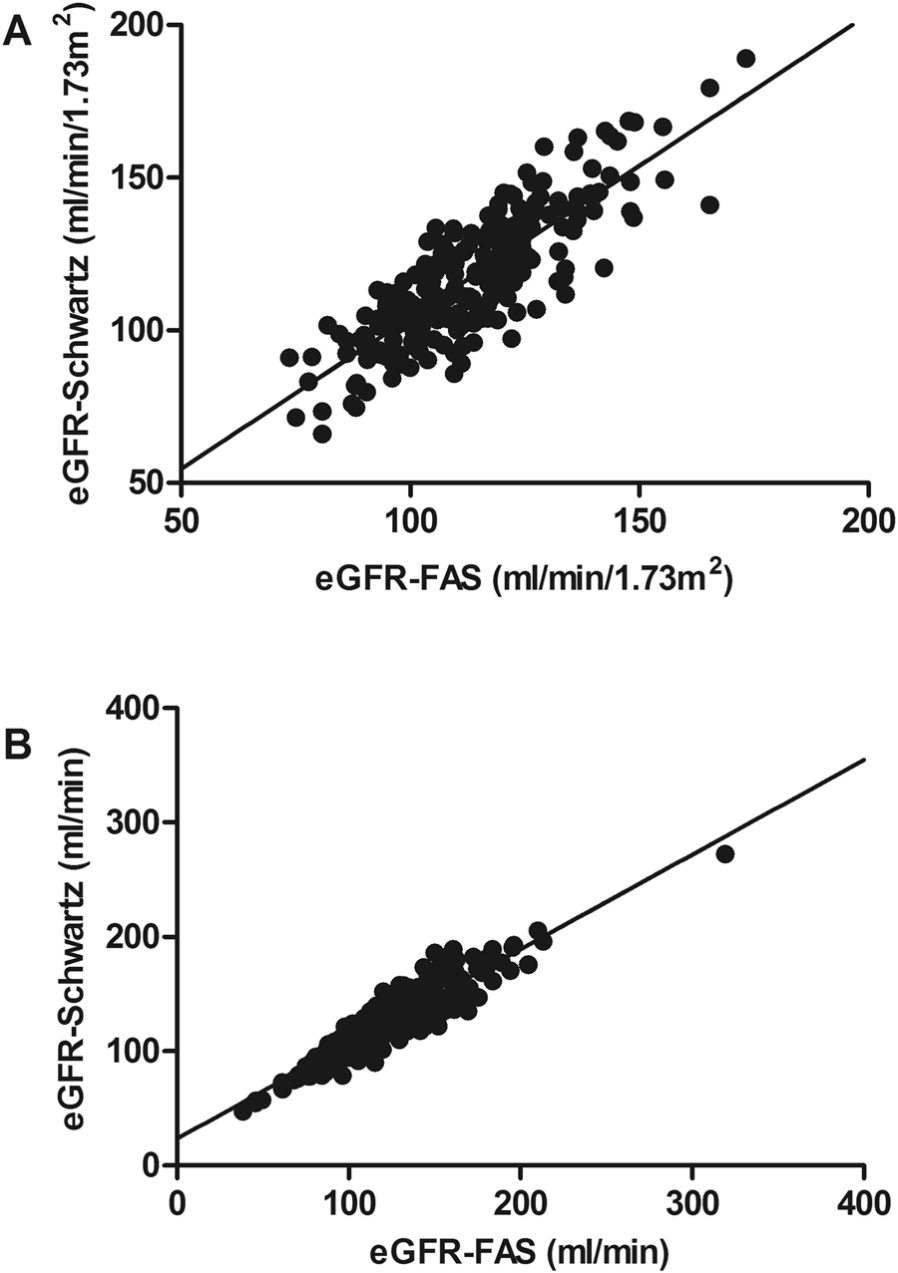
(**A**) Correlation between baseline eGFR-Schwartz and eGFR-FAS in ml/min/1.73 m2. Univariate linear regression model: y = 4.97 + 0.99* × with R^2^ = 0.685. (**B**) Correlation between baseline de-indexed eGFR-Schwartz and eGFR-FAS in ml/min. Univariate linear regression model: y = 23.74 + 0.83* × with R^2^ = 0.863.

At baseline, eGFR-FAS was positively correlated with eGFR-Schwartz (*r* = 0.828, *P* < 0.001, Fig. 2A), waist-to-hip ratio (*r* = 0.172, *P* = 0.010) and triacylglyceride concentration (*r* = 0.187, *P* = 0.003). No significant correlation with fasting glucose concentration or HOMA-IR was seen. De-indexed eGFR-FAS was positively correlated with de-indexed eGFR-Schwartz (*r* = 0.929, *P* < 0.001, Fig. 2B), age (*r* = 0.729, P < 0.001), BMI z-score (*r* = 0.388, P < 0.001), diastolic BP z-score (*r* = 0.190, *P* = 0.003), fasting glucose concentration (*r* = 0.189, *P* = 0.003), fasting insulin concentration (*r* = 0.514, *P* < 0.001), HOMA-IR (*r* = 0.531, *P* < 0.001) and triacylglyceride concentration (*r* = 0.210, *P* = 0.001). De-indexed eGFR-FAS was negatively correlated with the HDL-cholesterol concentration (*r* = −0.295, *P* < 0.001). De-indexed eGFR-Schwartz was correlated in the same manner as de-indexed eGFR-FAS with only minor differences in correlation coefficients.

### Longitudinal study

144 participants (58.8%) had a follow-up examination. Reasons for study discontinuation are displayed in Fig. 1. The mean interval between two assessments was 15.2 ± 3.9 months. Dropouts, as presented in Table 1, were significantly older than non-dropouts (13.0 ± 3.5 years versus 12.0 ± 3.0 years, *P* = 0.025) and had higher fasting glucose concentrations (4.1 mmol/L versus 4.0 mmol/L, *P* = 0.003) and lower total cholesterol concentrations (4.3 mmol/L versus 4.6 mmol/L, *P* = 0.03). eGFR-Schwartz and eGFR-FAS did not differ significantly nor did the serum creatinine/Q value ratio. Moreover, the percentage of participants with glomerular hyperfiltration did not differ significantly, except when the definition of an eGFR of more than 135 ml/min/1.73 m^2^ was used using the Schwartz equation.

Measurements before and after approximately one year lifestyle intervention as well as the mean of paired differences are presented in Table 2. BMI-z score, waist-to-hip ratio, diastolic BP z-score, HbA1c and concentrations of total cholesterol, LDL-cholesterol and triacylglyceride all decreased significantly after intervention. eGFR-Schwartz and eGFR-FAS decreased significantly as well. However, de-indexed eGFR-Schwartz and eGFR-FAS did not. De-indexed eGFR-FAS even increased significantly after intervention, as did the BSA, serum creatinine/Q value, fasting glucose concentration and the HOMA-IR.

**Table 2:** Comparison of pre- and post-intervention data of the children with at least one year of follow-up (n = 144).

Variable	Pre	Post	Mean of paired differences (pre-post)	*P-*value
Age (years)	12.0 ± 3.0	13.3 ± 3.0	−1.3 ± 0.3	<0.001*
BMI z-score	3.45 ± 0.65	3.28 ± 0.74	0.17 ± 0.44	<0.001*
BSA (m²)	1.81 ± 0.42	1.92 ± 0.38	−0.11 ± 0.10	<0.001*
Normal weight (%)	0.0	1.4	—	0.50
Overweight (%)	11.1	18.1	—	0.052
Obese (%)	48.6	43.8	—	0.28
Morbid obese (%)	40.3	36.8	—	0.30
Prepubertal (%)	28.9	18.0	—	0.06
Peripubertal (%)	26.3	36.9	—	0.38
Postpubertal (%)	44.7	45.0	—	0.50
Waist circumference z-score	5.5 (1.4–13.9)	5.3 (1.2–13.8)	0.1 (−5.8–4.2)	0.94
Hip circumference z-score	4.3 ± 1.9	4.4 ± 1.8	−0.1 ± 0.8	0.09
Waist-to-hip ratio	0.93 ± 0.07	0.91 ± 0.08	0.02 ± 0.08	0.04*
Systolic BP z-score	0.3 ± 1.0	0.1 ± 1.2	0.2 ± 1.3	0.09
Diastolic BP z-score	−0.4 ± 1.0	−0.7 ± 1.2	0.3 ± 1.2	0.01*
Serum creatinine (mg/dL)	0.55 ± 0.13	0.61 ± 0.13	−0.06 ± 0.07	<0.001*
eGFR-Schwartz (ml/min/1.73 m²)	119.6 ± 19.9	111.6 ± 18.3	8.0 ± 15.0	<0.001*
eGFR-Schwartz > 135 ml/min/1.73 m² (%)	22.2	11.8	—	0.004*
eGFR-Schwartz > 140 ml/min/1.73 m² (%)	15.3	7.6	—	0.03*
eGFR-Schwartz > 150 ml/min/1.73 m² (%)	6.9	2.8	—	0.15
eGFR-Schwartz de-indexed (ml/min)	123.5 ± 28.0	122.5 ± 25.6	0.9 ± 17.6	0.53
Serum creatinine (mg/dL)/Q value	0.96 ± 0.14	0.99 ± 0.13	−0.03 ± 0.12	0.003*
eGFR-FAS (ml/min/1.73 m²)	113.9 ± 16.3	110.1 ± 15.1	3.8 ± 15.1	0.003*
eGFR-FAS > 135 ml/min/1.73 m² (%)	13.0	5.6	—	0.03*
eGFR-FAS > 140 ml/min/1.73 m² (%)	6.9	4.2	—	0.34
eGFR-FAS > 150 ml/min/1.73 m² (%)	1.4	1.4	—	0.99
eGFR-FAS de-indexed (ml/min)	118.9 ± 30.5	122.4 ± 29.6	−3.5 ± 17.0	0.02*
UACR (mg albumin/g creatinine)	4.0 (0.0–36.2)	3.5 (0.0–401.3)	0.4 (−21.0–24.4)	0.86
Microalbuminuria (%)	2.1	4.3	—	0.45
Fasting glucose (mmol/L)	4.0 (2.5–5.6)	4.2 (2.5–5.3)	−0.2 (−1.3–1.9)	0.01*
Fasting insulin (pmol/L)	15.0 (2.0–111.2)	14.7 (2.1–64.8)	0.4 (−46.3–28.8)	0.19
HOMA-IR	2.5 (0.4–19.3)	2.7 (0.4–13.9)	−0.2 (−9.5–7.3)	0.03*
HbA1c (%)	5.3 ± 0.4	5.2 ± 0.3	0.2 ± 0.3	<0.001*
Total cholesterol (mmol/L)	4.6 ± 0.8	4.3 ± 0.8	0.3 ± 0.6	<0.001*
HDL-cholesterol (mmol/L)	1.2 ± 0.3	1.2 ± 0.3	0.0 ± 0.2	0.22
LDL-cholesterol (mmol/L)	2.8 ± 0.7	2.6 ± 0.7	0.2 ± 0.6	<0.001*
Triacylglycerides (mmol/L)	1.1 (0.4–3.7)	1.0 (0.4–3.8)	0.1 (−1.9–1.7)	0.04*

Abbreviations: BMI, body mass index; BSA, body surface area; BP, blood pressure; eGFR, estimated glomerular filtration rate; FAS, full age spectrum; UACR, urine albumin-to-creatinine ratio; HOMA-IR, homeostatic model assessment of insulin resistance; HbA1c, glycated hemoglobin; HDL, high density lipoprotein; LDL, low density lipoprotein.

Though, in the subgroup of participants with baseline glomerular hyperfiltration de-indexed eGFR changed significantly after intervention, depending on the definition for glomerular hyperfiltration and eGFR equation used; the results are displayed in Tables 3 and 4.

**Table 3:** Comparison of pre- and post-intervention eGFR-Schwartz stratified according to baseline glomerular hyperfiltration per definition.

Definition of glomerular hyperfiltration (n, percentile of 144 participants)	Pre eGFR-Schwartz (ml/min)	Post eGFR-Schwartz (ml/min)	Mean of paired differences (pre-post)	95% confidence interval	*P-*value
eGFR-Schwartz > 135 ml/min/1.73 m² (n = 32, 22.2%)	139.0 ± 32.1	128.1 ± 30.5	10.8 ± 20.6	3.4–18.3	0.006*
eGFR-Schwartz > 140 ml/min/1.73 m² (n = 22, 15.3%)	138.1 ± 32.0	128.8 ± 33.9	9.3 ± 15.4	2.5–16.1	0.010*
eGFR-Schwartz > 150 ml/min/1.73 m² (n = 10, 6.9%)	154.3 ± 28.4	140.9 ± 30.2	13.4 ± 14.6	3.0–23.8	0.017*
eGFR-FAS > 135 ml/min/1.73 m² (n = 18, 12.5%)	142.7 ± 29.2	129.3 ± 31.7	13.3 ± 15.8	5.5–21.2	0.002*
eGFR-FAS > 140 ml/min/1.73 m² (n = 10, 6.9%)	153.5 ± 29.4	142.6 ± 31.5	10.9 ± 19.2	−2.8–24.6	0.105
eGFR-FAS > 150 ml/min/1.73 m² (n = 2, 1.4%)	141.1 ± 8.9	126.9 ± 33.2	14.2 ± 24.3	n/a	n/a

Abbreviations: eGFR, estimated glomerular filtration rate; n/a, not applicable.

**Table 4:** Comparison of pre- and post-intervention eGFR-FAS stratified according to baseline glomerular hyperfiltration per definition.

Definition of glomerular hyperfiltration (n, percentile of 144 participants)	Pre eGFR-FAS (ml/min)	Post eGFR-FAS (ml/min)	Mean of paired differences (pre-post)	95% confidence interval	*P-*value
eGFR-Schwartz > 135 ml/min/1.73 m² (n = 32, 22.2%)	127.1 ± 31.0	119.2 ± 29.0	7.9 ± 16.9	1.8–14.0	0.013*
eGFR-Schwartz > 140 ml/min/1.73 m² (n = 22, 15.3%)	123.6 ± 28.3	117.2 ± 30.9	6.5 ± 13.4	0.5–12.4	0.035*
eGFR-Schwartz > 150 ml/min/1.73 m² (n = 10, 6.9%)	135.6 ± 24.1	126.3 ± 26.9	9.3 ± 14.8	−1.3–19.8	0.079
eGFR-FAS > 135 ml/min/1.73 m² (n = 18, 12.5%)	136.8 ± 30.6	126.0 ± 34.9	10.7 ± 16.1	2.8–18.7	0.011*
eGFR-FAS > 140 ml/min/1.73 m² (n = 10, 6.9%)	148.5 ± 32.6	140.5 ± 37.7	8.0 ± 19.6	−6.0–22.0	0.227
eGFR-FAS > 150 ml/min/1.73 m² (n = 2, 1.4%)	130.7 ± 9.1	116.5 ± 31.6	14.2 ± 22.5	n/a	n/a

Abbreviations: eGFR, estimated glomerular filtration rate; n/a, not applicable.

A subgroup that started the intervention as postpubertal adolescents also showed a significant decrease in de-indexed eGFR-Schwartz (mean paired difference 9.3 ± 14.9 ml/min, 95% confidence interval (CI) 4.0–14.5). De-indexed eGFR-FAS did not decrease significantly in this cohort (mean paired difference 3.7 ± 16.8 ml/min, 95% confidence interval (CI) −2.2–9.5).

Analysis of post-treatment parameters demonstrated a correlation between eGFR-FAS and eGFR-Schwartz (*r* = 0.726, *P* < 0.001), fasting insulin concentration (*r* = 0.171, *P* = 0.049) and triacylglyceride concentration *(r* = 0.184, *P* = 0.027*)*. De-indexed eGFR-FAS was positively correlated with de-indexed eGFR-Schwartz (*r* = 0.872, *P* < 0.001), age (*r* = 0.645, *P* < 0.001), BMI z-score (*r* = 0.419, *P* < 0.001), systolic BP z-score (*r* = 0.221, *P* = 0.008), diastolic BP z-score (*r* = 0.289, *P* = 0.001), fasting insulin concentration (*r* = 0.374, *P* < 0.001), HOMA-IR (*r* = 0.355, *P* < 0.001) and triacylglyceride concentration (*r* = 0.259, *P* = 0.001). De-indexed eGFR-FAS was negatively correlated with HDL-cholesterol concentration (*r* = −0.276, *P* = 0.001). De-indexed eGFR-Schwartz was correlated in the same manner as de-indexed eGFR-FAS with only minor differences in correlation coefficients. No associations of changes in de-indexed eGFR with changes in BMI z-score, blood pressure or parameters of glucose and lipid metabolism were found.

## Discussion

To the best of our knowledge, this prospective study is the first to evaluate the effect of a multidisciplinary lifestyle intervention on renal function in children and adolescents with overweight, obesity and morbid obesity without a history of renal disease. Depending on different definitions and on the equation used, glomerular hyperfiltration was present in 2% to 18% of the participants. In the total cohort of children, one year of lifestyle intervention did not significantly change eGFR after de-indexing for the BSA. However, in children with glomerular hyperfiltration at the start of the intervention, a significant decrease in eGFR was seen also after de-indexing eGFR. This decrease in eGFR was dependent on the eGFR equation and definition for glomerular hyperfiltration used.

Since measured GFR is lacking in this study, two different equations to estimate GFR were used. Though eGFR-Schwartz and eGFR-FAS correlated well, eGFR according to the Schwartz equation was approximately 5 ml/min/1.73 m^2^ higher compared to eGFR according to the FAS equation (see Fig. 2A). After intervention, de-indexed eGFR-Schwartz did not change significantly in the total group of children. De-indexed eGFR-FAS, however, increased significantly after intervention with a mean of 3.5 ml/min. Since the serum creatinine/Q value increases, it is plausible that this post-intervention increase in de-indexed eGFR-FAS can be explained by an increase in BSA.

All current eGFR equations have been designed for GFR indexed with BSA^8^. The convention of indexing GFR for BSA is routine practice since it allows direct comparison of GFR values between patients with different body sizes, and it helps in defining values that are considered as normal. Though indexation of the GFR by itself is theoretically questionable, in patients with unusual anthropometry the consequences of indexation GFR for the BSA is more important^9^. In case of obesity, indexation of GFR results in a substantially lower GFR than un- or de-indexed GFR. Another argument not to index GFR in case of obesity is that the number of nephrons does not increase with increased body fat, and by indexing GFR for BSA, one can ‘mask’ the hyperfiltrative state^9^. By using de-indexed eGFR in the longitudinal study bias due to possible weight, length or BSA change was avoided.

The pathogenesis of glomerular hyperfiltration in obesity is rather complex and not completely understood. It includes hormonal factors, including several adipokines, overactivity of the renin-angiotensin system and the renal sympathetic nervous system among others. Besides, insulin resistance has been independently associated with glomerular hyperfiltration. More specific, insulin resistance of podocytes is associated with lipid accumulation in the kidney, so called ‘fatty kidneys’^2^. In diabetes, glomerular hyperfiltration is mediated by the hyperglycaemic period over time^10^. In youth-onset type 2 diabetes, the most important risk factor for glomerular hyperfiltration is insulin resistance^11^.

In the present study, de-indexed eGFR was positively correlated with the BMI-z score and HOMA-IR. In case of high levels of glucose over time, the glucose concentration in the ultrafiltrate in the proximal tubule increases. This induces the expression of the sodium-glucose co-transporter 2 (SGLT2), which enhances reabsorption of glucose and sodium. The increased sodium reabsorption reduces the sodium concentration in the macula densa lining the distal tubule resulting in dilatation of the afferent arteriole and production of renin. Therefore, glucose might indirectly induce glomerular hyperfiltration^12^. Our group previously showed that hyperglycaemic glucose excursions are frequently observed in children with overweight and obesity^13^. Future studies should focus on the association between insulin resistance, the amount of time in a hyperglycaemic state and glomerular hyperfiltration in children and adolescents with overweight and obesity.

Almost 3% of the children presented with microalbuminuria at baseline, which is in line with other studies in children with obesity^14^. After lifestyle intervention, no difference in the percentages of children with microalbuminuria was noticed, which is in contrast to findings in obese adults where the urinary albumin to creatinine ratio decreased significantly as a result of weight loss^15,16^. This discrepancy might be due to an earlier, non-albuminuric phase of obesity-related renal damage present in children as compared to adults.

The study has some limitations. First, in this study, GFR was estimated using the Schwartz and FAS equation instead of directly measured. Alternative methods to estimate or determine renal function such as 24 hours urinary creatinine clearance, inulin or iohexol clearance were considered too invasive. Second, a random urine spot (RUS) was used for the estimation of the albumin excretion rate, whereas a 24 hours urine collection is considered the gold standard for the determination of albuminuria^17^.

Strengths of this study include the large study population and the prospective design. Moreover, because this outpatient lifestyle intervention program caused modifications in anthropometric, cardiovascular and metabolic parameters, it has been possible to correlate these alterations with changes in renal function.

In conclusion, this study demonstrates that in children and adolescents with overweight, obesity and morbid obesity with glomerular hyperfiltration, one year of lifestyle intervention leads to a significant decrease in eGFR. This finding, however, is dependent on the eGFR equation, the definition of glomerular hyperfiltration, and on whether eGFR is de-indexed using the body surface area. Further research should focus on the associated mechanisms and clinical implications.

## Methods

### Setting and study inclusion

Children and adolescents with overweight, obesity and morbid obesity were recruited from December 2010 to April 2014 from the Centre for Overweight Adolescent and Children’s Healthcare (COACH) at the Maastricht University Medical Centre. Within COACH, the health status of children with overweight and (morbid) obesity was evaluated, and children were treated and monitored as described previously^18^. Briefly, participation in the lifestyle intervention of COACH started with a comprehensive assessment aimed to exclude underlying syndromic or endocrine conditions of overweight, to evaluate complications and risk factors, and to gain insight in behaviour and (family) functioning. After the assessment, all children and their families were offered on-going, tailored and individual guidance with focus on lifestyle changes on a frequent basis at the outpatient clinic. Furthermore, participation in sports activities in groups and activities aimed at increasing nutritional knowledge were offered. A follow-up assessment including all the examinations performed during the initial assessment was offered annually to all children. Exclusion criteria for this study were secondary causes of overweight, pre-existent renal disease, current use of angiotensin converting enzyme inhibitors or angiotensin receptor blockers, psychomotor retardation, diabetes mellitus, cardiac disease and/or malignancies. Due to the continuous inflow of children in the COACH program, the moment of inclusion differed for each participant. The study was conducted according to the guidelines administered by the Declaration of Helsinki and approved by the medical ethical committee of the MUMC + . Informed consent was obtained from both participants and parents or legal guardian.

### Clinical assessment and anthropometry

All methods were carried out in accordance with relevant guidelines and regulations. A detailed history was taken from both participants and parents. In addition, all participants were subjected to a physical examination. Anthropometric data were collected while children were barefoot and wearing only underwear. Weight was determined using a digital scale (Seca, Chino, CA) and length was measured using a digital stadiometer (De Grood Metaaltechniek, Nijmegen, the Netherlands). Overweight, obesity and morbid obesity were defined according to the International Obesity Task Force criteria^19^. Waist circumference was measured with a non-elastic measuring tape at the end of a natural breath at midpoint between the top of the iliac crest and the lower margin of the last palpable rib. Hip circumference was measured with a non-elastic measuring tape at the maximum circumference over the bottom. Waist and hip circumference z-score was determined according the Dutch reference values for children^20^. Puberty stage was determined by the physicians using the Tanner G/M stadia^21,22^. When no Tanner stadium was reported, girls > 14.3 years and boys > 15.0 years were classified as Tanner G/M stage 4. Tanner stages were subsequently divided in three subgroups: prepubertal (Tanner G/M stage 1), peripubertal (Tanner G/M stage 2–3) and postpubertal (Tanner G/M stage 4–5)^23^.

### Metabolic measurements and analyses

Venous blood samples were collected after a minimum of 8 hours overnight fasting. Fasting plasma glucose concentrations and serum total cholesterol, low-density lipoprotein (LDL) cholesterol, high-density lipoprotein (HDL) cholesterol, and triacylglyceride concentrations were determined with the Cobas 8000 modular analyser (Roche). Serum insulin concentrations were analysed with the Immulite-1000 (Siemens Healthcare Diagnostics). Glycated haemoglobin (HbA1c) concentrations were determined with the fully automated HPLC Variant II (Bio-Rad Laboratories). After obtaining the fasting blood sample an oral glucose tolerance test (OGTT) was performed. 1.75 grams of glucose per kilogram of bodyweight was dissolved into 200 mL water, with a maximum of 75 grams of glucose in total, and given orally. Plasma blood glucose concentrations were measured every thirty minutes during two hours. Creatinine was measured in serum by means of spectrometry (Cobas 8000 modular analyser series, Roche Diagnostics USA).

### GFR estimation and defining glomerular hyperfiltration

Glomerular filtration rate (GFR) was estimated by means of the equation by Schwartz *et al*.^24^: estimated GFR (eGFR; ml/min/1.73 m^2^) = 0.413 × height (cm)/serum creatinine (mg/dL)). Moreover, since serum creatinine in children depends on age and gender, and the aim of this study was to evaluate serum creatinine independent of age and gender, serum creatinine of individual subjects was normalized with the median serum creatinine value at the corresponding age and gender. So, we also estimated GFR using the Full Age Spectrum (FAS) equation, proposed and validated by Pottel *et al*.: eGFR = 107.3/(serum creatinine (mg/dL)/Q), in which Q is the median value of the serum creatinine defined according gender and age^8,25^. Since all current eGFR equations have been designed for GFR indexed with body surface area (BSA)^8^, eGFR was de-indexed using the BSA. BSA was estimated using the Haycock equation: BSA = 0.02465 × Wt ^0.5378^ × Ht ^0.3964^ (with Wt as weight in kilograms and Ht as height in centimetres)^26,27^. De-indexed eGFR was then calculated by multiplying eGFR values by BSA and dividing by 1.73 m^2^.

For defining glomerular hyperfiltration, several cut-off values are used^28^. Therefore, in this study, different cut-off values were used for stratification: an eGFR of > 135 ml/min/1.73 m^2^, according to the 90^th^ percentile of a cohort described by Piepsz *et al*^29^., an eGFR > 140 ml/min/1.73 m^2^ ^30^ and an eGFR > 150 ml/min/17.3 m^2^ ^31^, based on reference values of Pottel *et al*.^32^: a median GFR of 107.3 mL/min/1.73 m^2^ with a standard deviation of 21.5 mL/min/1.73 m^2^ leads to a 97.5th percentile of 150 mL/min/1.73 m^2^. The amount of microalbuminuria was estimated by means of a random urine spot (RUS). Albumin was measured in a quantitative manner using a immunoturbidimetric method and the urine albumin-to-creatinine ratio (UACR) was calculated by dividing the albumin concentration by the creatinine level. Exact determination of the UACR was not possible <1.77 mg/g. Microalbuminuria was defined as an UACR of ≥30 mg/g and ≤300 mg/g^33^.

### Blood pressure

Daytime blood pressure was measured approximately 20 times with an interval of three minutes between each measurement using the Mobil-O-Graph (I.E.M. GmbH, Stolberg, Germany). The cuff size was fitted according the circumference of the upper arm. Mean blood pressure was calculated and systolic and diastolic blood pressure z-scores were determined according to reference values related to height and gender^34^.

### Statistical analyses

Statistical analyses were performed with IBM SPSS Statistics Version 20.0. The Shapiro-Wilk test was used to test normality and Levene’s test was used to test for the equality of variances. Normally distributed data are presented as mean ± standard deviation, whereas skewed data are reported as median (minimum – maximum). Comparisons between different groups were made with Pearson’s chi-square test, Fisher’s exact test, the independent t-test, the Mann-Whitney U test, the paired-samples t-test, the Wilcoxon signed-rank test and the McNemar test as appropriate. All *P*-values are two-tailed and a *P*-value below 0.05 was considered statistically significant.
